# Patients living with HIV have quantitatively inadequate food consumption

**DOI:** 10.20945/2359-3997000000623

**Published:** 2023-05-29

**Authors:** Bárbara Ferreira Vercesi, Izabela Spereta Moscardini, Gabriel Perri Esteves, Rebeca Antunes Beraldo

**Affiliations:** 1 Universidade de São Paulo Faculdade de Medicina de Ribeirão Preto Departamento de Clínica Médica Ribeirão Preto SP Brasil Universidade de São Paulo, Faculdade de Medicina de Ribeirão Preto, Departamento de Clínica Médica, Ribeirão Preto, SP, Brasil; 2 Universidade de São Paulo Faculdade de Medicina Divisão de Reumatologia São Paulo SP Brasil Universidade de São Paulo, Faculdade de Medicina, Divisão de Reumatologia, Grupo de Pesquisa em Fisiologia Aplicada e Nutrição; Escola de Educação Física e Esporte, São Paulo, SP, Brasil

**Keywords:** HIV, energy expenditure, food consumption, lipodystrophy

## Abstract

**Objective:**

The objective of the current study was to estimate energy expenditure and compare it with the usual food consumption of PLWH, and to determine whether duration of high-potency antiretroviral therapy (HAART) influenced nutritional intake and adequacy.

**Materials and methods:**

Anthropometric measurements and bioelectrical impedance analysis (BIA) provided data for estimating resting energy expenditure (REE) using Melchior's equations. Dietary Reference Intakes (DRIs) and 24-Hour Recall were used to verify if reported food intake aligned with energy, macro and micronutrient recommendations.

**Results:**

Sixty one patients with a mean age of 52 ± 9.4 years and who had a high frequency of diabetes mellitus (24.5%), hypertension (54%), and dyslipidemia (90.1%) were evaluated. Estimated REE of female and male patients with less than 10 years of HAART was 1791 (1717.5; 1887.2) and 1941 (1808; 2335.6), and their estimated energy intake was 900.5 (847;1221.9) and 2095.4 (1297.5; 2496.4), respectively. The estimated REE for female and male patients with more than 10 years of HAART was 1796.20 (1598.9;1820.7) and 2105 (1913.4; 2308), and their estimated energy intake was 1566 (1353.1; 1764.3) and 1999.7 (1706.5; 2508.1), respectively. Being on HAART for more than 10 years was associated with increased energy intake (533 (95% CI 3; 1063) kcals), but not with meeting energy requirements.

**Conclusion:**

Patients had an atherogenic metabolic profile, inadequate dietary pattern, and a similar REE, regardless of HAART duration, contributing even more to the increased risk of cardiovascular diseases.

## INTRODUCTION

The interaction of high potency antiretroviral therapy (HAART) with the inflammation and infection generated by human immunodeficiency virus (HIV) itself is associated with serious unwanted effects, such as metabolic changes and abnormal redistribution of body fat. This set of alterations is called HIV-associated lipodystrophy syndrome (1). Abnormal redistribution of body fat (lipodystrophy) is the main feature of this syndrome, being characterized by loss of subcutaneous fat mainly in the face, gluteal region and limbs (lipoatrophy), and accumulation of fat in the cervical back, breasts and visceral (lipohypertrophy). This modulation of fat can be accompanied by insulin resistance and dyslipidemia, which are features of the metabolic syndrome, and involves a set of cardiovascular disease risk factors, including hypertension, low levels of high-density lipoprotein cholesterol levels, hypertriglyceridemia, and central obesity (2).

Given that HAART is associated with several adverse metabolic effects, questions have arisen regarding the energy expenditure of people living with HIV/AIDS (PLWHA) with HAART-associated lipodystrophy (HAL) (3). A study suggested that resting energy expenditure (REE), *i.e.*, the rate at which the body consumes energy while inactive, was considerably higher in HIV-infected patients with HAART-associated lipodystrophy than in infected patients who did not develop lipodystrophy. An increased food and fat intake has also been linked to patients with HAL (4). When estimating energy expenditure in clinical practice, it is more feasible to use predictive equations developed through validation studies. These equations utilize variables that are close correlated to REE, and are evaluated by being compared to a more accurate method. Melchior's equations, which were developed specifically for individuals living with HIV, are the best option for the assessment of energy expenditure in these patients (5).

Thus, the objective of the present study was to calculate the REE and total energy expenditure (TEE) using these predictive equations that are most suitable for this population. Habitual food consumption was also assessed, and used to verify whether energy and macronutrient recommendations were met or exceeded, which could exacerbate atherogenic profile and cardiovascular risk in patients with lipodystrophy syndrome. We also aimed to identify whether the duration of HAART treatment influenced energy intake.

## MATERIALS AND METHODS

This was a cross-sectional analytical study carried out at the Hospital of Ribeirão Preto Medical School (HC-FMRP). This study adheres to the STrengthening the Reporting of OBservational studies in Epidemiology (STROBE) checklist (Supplementary Material 1). Sixty-one outpatients from the Specialized Unit for the Treatment of Infectious Diseases (*Unidade Especial de Tratamento de Doenças Infecciosas* – UETDI) were randomly selected (for a flowchart diagram, see Supplementary Material 2). The study was approved by the research ethics committee of the institution (number: 2.485.545). Patients on outpatient follow-up who met the selection criteria were invited to participate in the research project and signed an informed consent form. Inclusion criteria were: positive serology for HIV, age between 18 and 65 years, being on HAART for at least 6 months, being clinically stable and presenting metabolic syndrome (6).

### 24-hour dietary recall

The usual diet was characterized by the application of three 24-hour dietary recalls by trained interviewers. For the calculation of nutrients, Microsoft Excel software was used, through planned spreadsheets with data from the Brazilian Table of Food Composition – TACO (7). Usual dietary intake was estimated with The Multiple Source Method (MSM), a program which calculates the usual dietary intake from 24-hour recalls using statistical methods, aiming to reach a more accurate estimate of individual values (8). Averages of these results were compared to the estimated average requirement (EAR), a reference value that corresponds to the median of the distribution of the requirement of a given nutrient in a group of healthy individuals with the same sex and stage of life, sufficient to meet the nutrient requirements of 50% of the population, present in the DRIs (dietary reference intakes) (9). Optimal intake of cholesterol, trans fatty acids, and saturated fatty acids were considered to be as low as possible while consuming a nutritionally adequate diet (10).

### Anthropometric measurements and calculation of indices

Anthropometric measurements were performed at the same meeting. Body weight, in kilograms, was measured using a platform-type Filizola electronic scale, with a maximum capacity of 300 kg and accuracy of 0.1 kg. Height was measured with a stadiometer with an accuracy of 0.1 cm. Body mass index (BMI) was calculated as weight (kg) divided by height (m) squared. Circumference measurements were performed using a Sanny metallic tape measure, with a precision of 0.1 cm and a maximum length of 2 m. The assessment of abdominal circumference (AC) was performed at the midpoint between the last rib and the iliac crest in a horizontal plane. Waist circumference (WC) was measured with the individual standing, using an inextensible measuring tape with graduations up to 150 cm and a minimum of 0.1 cm; the measurement was taken at the midpoint between the last rib and the iliac crest. The hip circumference (HC) was measured in the region with the greatest circumference between the abdomen and the thigh (11). The waist-hip ratio (WHR) was calculated by dividing the WC (cm) by the HC (cm) and the waist-thigh ratio (WTR) by dividing the WC (cm) by the thigh circumference (TC) (cm). The World Health Organization (WHO) reference values were used to classify risk of metabolic alterations (12).

### Biochemical analyses

Serum lipid enzyme levels (total cholesterol = TC, triglycerides = TG and high-density lipoprotein = HDL) were measured by the colorimetric method with the CONTEGRA 40 instrument (Roche Diagnostics, IN, USA). Low density lipoprotein (LDL) was calculated using the Friedewald formula [LDL CT - (TG5 + HDL)]. Glucose was determined on an automatic analyzer (230, Yellow Springs Instruments Inc., Yellow Springs, OH, USA). The following were considered: TC ≥ 220 mg/dL, TG ≥ 150 mg/dL, HDL ≤ 40 mg/dL, LDL ≥ 130 mg/dL or treatment for dyslipidemia; fasting glucose ≥ 100 mg/dL or on treatment (6).

### Bioelectrical impedance analysis (BIA)

After removing excess clothes and jewellery, the individual assumed a supine position. The skin of the right hand and foot was sanitized with 70% alcohol and, after drying, the procedures began. The distal electrodes (current electrodes) were fixed on the anterior surface of the foot, on the final distal part of the second metatarsal and in the posterior area of the hand, on the final distal part of the third metacarpal. The proximal electrodes (reading electrodes) were placed on the prominence of the radius and ulna on the posterior surface of the wrist, and the other electrode between the malleolus of the tibia and fibula on the anterior surface, at the junction between the foot and leg. Subsequently, using the RJL SYSTEM^®^ device, a current of 50kHz was applied and the values of R and Xc in Ohm were determined to calculate fat mass and fat-free mass using the Kotler equation (13).

### Predictive equations for calculating REE

The best equations for estimating REE are population-specific, such as Melchior's, which was based on a sample of 129 people living with HIV without the presence of secondary infections, which matches the population of the present study (5). The selected Melchior equation is as follows: REE (kj/day) = 1379 + 123 x lean mass (kg) (14). The energy expenditure obtained was multiplied by the activity factor of 1.2, recommended for sedentary individuals/practitioners of light physical activity, which closely matches the level of physical activity of participants in our sample.

### Statistical analysis

Data are presented as mean and standard deviation for demographic, anthropometric and body composition variables, and median alongside interquartile range (p25 – p75) for nutritional variables. Participants were separated according to the duration of HAART into < 10 years of HAART or ≥ 10 years of HAART. Baseline demographic variables between these groups were compared using Student's t test or the nonparametric Wilcoxon test for continuous variables, and the chi-square test for categorical variables.

To assess whether the duration of HAART treatment associated with current energy intake, linear regression models were used, with energy intake considered as a dependent variable, and duration of HAART (< 10 years or ≥ 10 years) as the independent variable. Additionally, to assess whether HAART treatment associated with the proportion of patients that met their energy requirements, logistic regression models were also used, this time with the classification of whether the patient met their energy requirements (“Yes” or “No”) considered as the dependent variable, and duration of HAART (< 10 years or ≥ 10 years) as the independent variable. All models were adjusted for sex, age and body weight. A significant result was assumed when p < 0.05. All analyses were done using R and Rstudio software (R version 4.2.1, R Core Team, Vienna, Austria).

## RESULTS

The final study sample consisted of 61 patients, with a mean age of 52 (±9.4) years, mean time of infection of 15 (±7.1) and mean time of treatment with HAART of 14 (±7). The mean age of patients with less than 10 years of HAART use was 52.5 ± 10.3, and they had a percentage of 13% of diabetes mellitus, 43% of hypertension and 82% of dyslipidemia. Regarding medication use, 17% used antidiabetic drugs, 47% antihypertensive drugs and 39% lipid-lowering drugs. In contrast, patients with longer use of HAART (more than 10 years), had a mean age of 52.5 ± 7.18, and higher presence of diabetes mellitus (31.5%), hypertension (60%) and dyslipidemia (94%). Antidiabetic drugs were used by 24.5% of this group, antihypertensive drugs by 55.7% and lipid-lowering drugs by 36% (Table 1).

**Table 1:** Profile of HIV-positive people on HAART treated at the UETDI outpatient clinic, 2020

Variable	<10 Years HAART (n = 23)	≥10 Years HAART (n = 38)	Total (n = 61)
Age	52.5 ± 10.3	52.5 ± 7.1	52 ± 9.4
Positive serology time	7.5 ± 6.7	18 ± 5.5	15 ± 7.1
Treatment time	7 ± 2.4	18 ± 5.6	14 ± 7
Diabetes mellitus (n and % of individuals)	3 (13%)	12 (31.7%)	15 (24.5%)
Hypertension (n and % of individuals)	10 (43%)	23 (60%)	33 (54%)
Dyslipidemia (n and % of individuals)	19 (82%)	36 (94%)	55 (90.1%)
Use of antidiabetic drugs (n and % of individuals)	4 (17%)	11 (28%)	15 (24.5%)
Use of antihypertensive drugs (n and % of individuals)	11 (47%)	23 (60%)	34 (55.7%)
Use of lipid-lowering drugs (n and % of individuals)	9 (39%)	13 (34%)	22 (36%)

No statistically significant difference between groups (all p > 0.05).

All measurements for female participants were above reference values, indicating the presence of obesity and substantially higher cardiovascular risk. BMI in the female group that had been using HAART for more than 10 years was similar to the group that had been on treatment for less time, but the smaller hip and thigh circumferences may point to the development of lipodystrophy. Male patients also presented measures that exceeded the reference values. However, in the group that used HAART for more than 10 years, the cardiovascular risk was substantially higher, as indicated by the values of waist circumference and waist-hip ratio (higher than the other male group) (Table 2) (12). BIA indicated that lean mass and fat mass values were similar in both groups (Table 3).

**Table 2:** Measures and anthropometric indices distributed by sex and duration of HAART of people living with HIV treated at the UETDI outpatient clinic, 2020

Variable	<10 Years HAART (n = 23)		≥10 Years HAART (n = 38)	
	Female	Male	Female	Male
	(n = 9)	(n = 14)	(n = 18)	(n = 20)
Weight (kg)	84.4 ± 18.5	86.6 ± 22.7	84.3 ± 17.3	89.9 ± 20.6
Waist circumference (cm)	113.5 ± 12.8	101 ± 18.4	101.2 ± 24.3	108 ± 15.9
Hip circumference (cm)	117 ± 14.2	106 ± 9.9	108.5 ± 25.7	104.5 ± 13.1
Thigh circumference (cm)	63 ± 7.3	52 ± 10.3	56.7 ± 7,1	56 ± 7
Waist-hip ratio	0.9 ± 0.03	0,9 ± 0.06	0.9 ± 0.06	1 ± 0.06
Waist-thigh ratio	1.7 ± 0.09	1.9 ± 0.1	1.7 ± 0.3	1.9 ± 0.1
BMI	32.2 ± 7.4	29.2 ± 8.3	31.7 ± 6.6	30.1 ± 6.4

BMI: body mass index, reference value: >24.9 kg/m^2^; WC: waist circumference, female reference value ≥ 80 cm (increased risk) and ≥ 80 cm (substantially increased risk) and male ≥ 94 cm (increased risk) and ≥ 102 cm (substantially increased risk); WHR: waist-hip ratio, female reference value ≥ 0.85 and male ≥ 1; All p < 0.05.

**Table 3:** Body composition obtained by analyzing the total bioelectrical impedance of people living with HIV on HAART treated at the UETDI outpatient clinic, 2020

Variable	<10 Years HAART (n = 23)		≥10 Years HAART (n = 38)	
	Female	Male	Female	Male
	(n = 9)	(n = 14)	(n = 18)	(n = 20)
Lean mass (kg)	49.5 ± 6.5	57.4 ± 11.7	51 ± 8.8	62.8 ± 10.8
Fat mass (kg)	37.3 ± 12.3	24.9 ± 13.4	29.5 ± 10.5*	25.1 ± 12.1
Body fat percentage (%)	41.3 ± 14.2	28.2 ± 9.5	36.2 ± 6.3	29.2 ± 8

* p < 0.05

**Figure 1:**
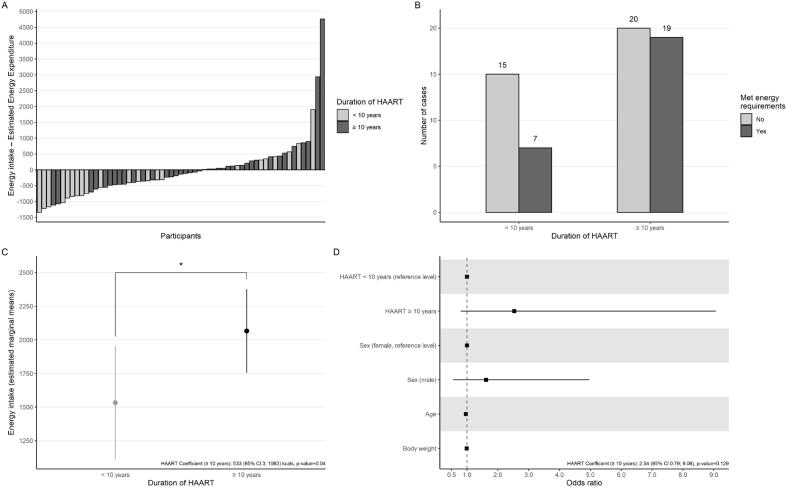
Energy intake status and the role of duration of HAART in people living with HIV treated at the UETDI outpatient clinic. **(A)** Values for the total energy intake minus the estimated energy requirements from each individual in the sample; **(B)** number of cases that met estimated energy requirements according to duration of HAART; **(C)** results from the linear regression model (estimated marginal means, or difference between groups) showing increased energy intake among individuals under HAART for 10 years or more; **(D)** results from the logistic regression models showing odds ratio alongside 95% confidence intervals, with no statistically significant association between duration of HAART and odds of meeting energy requirements. Models were adjusted for sex, age and body weight. *: p < 0.05. HIV: human immunodeficiency virus; HAART: high-potency antiretroviral therapy; UETDI: Specialized Unit for the Treatment of Infectious Diseases.

Regarding food consumption, 35 individuals presented with an energy intake (kcal) below their recommendation, 15 (68%) being on HAART for less than 10 years, and 20 (51%) being on HAART for more than 10 years (Figure 1, panels A and B). Linear regression models showed that being on HAART for 10 or more years was significantly associated with higher energy intake then being on HAART for less than 10 years (Coefficient: 533 (95% CI 3; 1063) kcals, p-value = 0.04). However, being on HAART for longer duration was not associated with increased or decreased odds of having adequate energy intake (Coefficient: 2.54 (95% CI 0.79; 9.06) odds ratio, p-value = 0.129) (Figure 1, panels C and D). Average total energy intake was below the DRIs recommendations in both groups, while the percentages of lipids, proteins and carbohydrates were within the recommended range. Both groups presented, on average, inadequate ingestion of fiber and micronutrients such as potassium, iron, calcium and vitamin C, with the exception of sodium (above recommended ingestion) and zinc (adequate) (Table 4).

**Table 4:** Composition of food consumption of people living with HIV on HAART treated at the UETDI outpatient clinic, 2020

Variable	<10 Years HAART (n = 23)	Consumption	≥10 Years HAART (n = 38)	Consumption
Energy (kcal)	1507 (1019.2; 2162.3)	↓	1750.6 (1552; 2073.6)	↓
Carbohydrates (g)	160.9 (103.9; 306.3)		231.4 (172.6; 312.5)	
Proteins (g)	84.8 (68.4; 109.1)		81.5 (67; 103.4)	
Lipids (g)	51.4 (39.2; 68.6)		53 (40.2; 70.8)	
Fibers (g)	14.8 (11.8; 31.0)	↓	21.3 (14.9; 30.0)	↓
Cholesterol (mg)	220.7 (159.3; 374.5)		205.3 (156.1; 351.7)	
Saturated fat (g)	14.6 (10,1; 24.1)		16.4 (12.8; 22.2)	
Monounsaturated fat (g)	18.9 (11.4; 19.9)		16.5 (13.1; 23.2)	
Polyunsaturated Fat (g)	15.3 (12.8; 18.5)		15.5 (11; 17.8)	
Omega 6 (g)	13.1 (11.6;16.9)		13.7 (9.9; 16.2)	
Omega 3 (g)	1.4 (1.1; 1.7)		1.4 (1.1; 1.6)	
Trans fat (g)	0.7 (0.4; 1.6)		0.9 (0.6; 2.2)	
Calcium (mg)	203 (89.1; 451.6)	↓	370.4 (198.5; 589.9)	↓
Iron (mg)	6.7 (5.4; 10.9)	↓	7.1 (5.9; 10)	↓
Sodium (mg)	1693.6 (796.3; 2322.1)	↑	1531.9 (1146.2)	↑
Potassium (mg)	2403.7 (1698.1; 2836.7)	↓	2648.9 (2193.8; 3332.9)	↓
Zinc (mg)	11.5 (8.3; 14.9)	Adequate	8,8 (6.3; 13.5)	Adequate
Vitamin C (mg)	15 (4.1; 21.9)	↓	41.9 (17.2; 96)	↓

No statistically significant difference between groups (all p > 0.05).

When separating individuals according to sex, in males, estimated energy expenditure was higher in patients who had been using HAART for more than 10 years compared to men who had been on treatment for less time. In women, there was a slight increase in estimated energy expenditure in the group with longer use of HAART (Table 5).

**Table 5:** Energy expenditure, food intake and their comparison distributed by sex and time of HAART use among people living with HIV treated at the UETDI outpatient clinic, 2020

Variable	<10 Years HAART (n = 23)		≥10 Years HAART (n = 38)	
	Female (n = 9)	Male (n = 14)	Female (n = 18)	Male (n = 20)
Energy expenditure (kcal)	1791 (1717.5; 1887.2)	1941 (1808; 2335.6)	1796.2 (1578.9; 1820.7)	2105 (1913.4; 2308)
Food intake (kcal)	900.5 (847; 1221.9)	2095.4 (1297.5; 2496.4)	1567 (1353.1; 1764.3)	1999.7 (1706.5; 2508.1)
Intake above energy expenditure (n and % of individuals)	2 (22.2%)	7 (50%)	7 (38,8%)	9 (45%)
Food intake below energy expenditure (n and % of individuals)	7 (77.8%)	7 (50%)	11 (61,2%)	11 (55%)

No statistically significant difference between groups (all p > 0.05).

## DISCUSSION

A quantitatively inadequate dietary profile, high prevalence of metabolic alterations, and redistribution of body fat was observed in this group of PLWH using HAART. Statistical models showed that individuals on HAART for 10 years or longer consumed more kcals than their counterparts, even after adjusting for important confounding variables such as sex, age and body weight. HAART duration, however, was not associated with odds of having an adequate energy intake relative to individual needs, suggesting that although HAART may impact energy intake, this impact may be relatively small.

Although the food consumption evaluation revealed that some nutrients had similar intakes in both groups, some were different, such as energy, carbohydrates, lipids, fiber, calcium, sodium, potassium and vitamin C, indicating that patients with more than 10 years on HAART have a higher food intake. When considering the average of the entire group, insufficient intake of energy, some micronutrients (potassium, iron, calcium and vitamin C) and fiber, in addition to a high consumption of sodium, was observed in both groups, when compared to DRIs (9). A study with a similar sample to ours carried out by Duran and cols. (2008), showed an inadequate dietary pattern and a high prevalence of risk factors related to the development or progression of cardiovascular diseases in these patients, which, alongside our results, reinforces the conclusion that PLWH using HAART are likely to present with unhealthy diets (15).

No considerable differences were observed in the resting energy expenditure of our sample between men and women of the two groups who used HAART for more or less than 10 years. In contrast, in the study by Sutinen and Yki-Järvinen (2007), differences were observed in a group that used HAART without lipodystrophy (HAART-LD) when it was compared to another that also used HAART, but that presented with lipodystrophy (HAART+LD). The HAART+LD group presented a higher energy expenditure and a higher food intake, which was expected in view of the higher energy expenditure (4). This may help explain our findings. Since most patients in our sample presented lipodystrophy, their energy expenditure was similar across both groups, regardless of the time they had been on HAART. Additionally, logistic regression showed that duration of HAART did not influence the odds of reaching energy recommendations, despite HAART affecting energy intake on average. This suggests that although longer durations of HAART use may be associated with increased energy intake, this increase is not sufficient to lead to adequate energy intake.

It is undeniable that HAART has brought several advantages to patients who use it, but also that its use may be linked to metabolic changes and with the consequent development of diabetes mellitus, hypertension and dyslipidemia. This was observed in our study, where we found a higher prevalence of patients with longer time on HAART who had these comorbidities compared to those who used the therapy for less time. The same happened in the study by Tasca and cols. (2021), which evaluated 94 patients in São Paulo, and reported that patients who used antiretroviral drugs, especially those who used them for a longer time, developed metabolic and cardiac alterations (16). Such alterations can be explained by the fact that adipose tissue is one of the main factors for the development of metabolic alterations and chronic diseases, and its redistribution is the focus of lipodystrophic syndrome (17). The association between lipodystrophy and HAART exists because protease inhibitors impair adipocyte differentiation through mechanisms that include the inhibition of proteins that are part of lipid metabolism and insulin dysregulation, resulting in adipose hypertrophy (especially in the viscera), decreased high-density lipoprotein (HDL), increased triglycerides, insulin resistance, type 2 diabetes mellitus, and hypertension (18).

These factors can lead to the presence of metabolic syndrome. This syndrome was found in about half of the sample of a study by Vargas-Pacherrez and cols. (19), which estimated the presence of metabolic syndrome and associated factors in a group of people living with HIV on antiretroviral therapy. This parameter was also evaluated within our study, but no influence of time of HAART use was observed. We found high values of waist, hip and thigh circumference in both groups, regardless of the duration of HAART. However, when separated according to sex, men with longer duration of HAART exhibited a considerably higher cardiovascular risk, according to the values of waist circumference and waist-hip-ratio. Studies suggest that metabolic syndrome in HIV is also influenced by the use of antiretroviral drugs, which consequently means that these patients will have a greater accumulation of visceral fat (20). These alterations are characteristic of an atherogenic metabolic profile, which may further increase the risk of developing cardiovascular disease (17).

The main strength of our study was that it evaluated the food intake of patients and compared it with their energy expenditure, since it is known that, regardless of HIV infection, an inadequate dietary pattern is associated with risk factors for the development of cardiovascular diseases (21). In addition, although it does not have the power to completely remove all errors, the MSM program is a useful and free tool to minimize intrapersonal variance, and generate data that are most representative possible from the usual diet.

Our study also has some limitations. We acknowledge that there are other factors that may influence the assessment of food intake that were not addressed in this study, including an underreporting of energy intake in repeated 24-hour recalls, and the presence of metabolic syndrome as an inclusion criterion, since the onset of the syndrome is usually associated with an unhealthy dietary pattern. Future studies should continue investigating the influence of varying durations of HAART use on metabolic and anthropometric parameters, in addition to food intake and energy expenditure, in order to verify these findings.

In conclusion, patients within the current study showed an atherogenic metabolic profile, an inadequate dietary pattern, and a similar REE, regardless of duration of HAART, which may exacerbate cardiovascular disease risk in PLWH. Thus, treatment for human immunodeficiency syndrome should not be carried out in isolation with the use of drugs such as antiretroviral drugs, but in conjunction with the adoption of a healthy, balanced and nutritionally adequate diet, in addition to the practice of physical activities.

## SUPPLEMENTARY MATERIAL 1

**Table:** STROBE Statement – Checklist of items that should be included in reports of cohort studies

	Item No	Recommendation	Yes, No, Not applicable
Title and abstract	1	(a) Indicate the study's design with a commonly used term in the title or the abstract	Yes
		(b) Provide in the abstract an informative and balanced summary of what was done and what was found	Yes
**Introduction**			
Background/rationale	2	Explain the scientific background and rationale for the investigation being reported	Yes
Objectives	3	State specific objectives, including any prespecified hypotheses	Yes
**Methods**			
Study design	4	Present key elements of study design early in the paper	Yes
Setting	5	Describe the setting, locations, and relevant dates, including periods of recruitment, exposure, follow-up, and data collection	Yes
Participants	6	(a) Give the eligibility criteria, and the sources and methods of selection of participants. Describe methods of follow-up	Yes
		(b) For matched studies, give matching criteria and number of exposed and unexposed	Not applicable
Variables	7	Clearly define all outcomes, exposures, predictors, potential confounders, and effect modifiers. Give diagnostic criteria, if applicable	Yes
Data sources/measurement	8*	For each variable of interest, give sources of data and details of methods of assessment (measurement). Describe comparability of assessment methods if there is more than one group	Yes
Bias	9	Describe any efforts to address potential sources of bias	
Study size	10	Explain how the study size was arrived at	Yes
Quantitative variables	11	Explain how quantitative variables were handled in the analyses. If applicable, describe which groupings were chosen and why	Yes
Statistical methods	12	(a) Describe all statistical methods, including those used to control for confounding	Yes
		(b) Describe any methods used to examine subgroups and interactions	Yes
		(c) Explain how missing data were addressed	Yes
		(d) If applicable, explain how loss to follow-up was addressed	Not applicable
		(e) Describe any sensitivity analyses	Yes
**Results**			
Participants	13*	(a) Report numbers of individuals at each stage of study – *e.g.* numbers potentially eligible, examined for eligibility, confirmed eligible, included in the study, completing follow-up, and analysed	Yes
		(b) Give reasons for non-participation at each stage	Yes
		(c) Consider use of a flow diagram	Yes
Descriptive data	14*	(a) Give characteristics of study participants (eg demographic, clinical, social) and information on exposures and potential confounders	Yes
		(b) Indicate number of participants with missing data for each variable of interest	Not applicable
		(c) Summarise follow-up time (*e.g.*, average and total amount)	
Outcome data	15*	Report numbers of outcome events or summary measures over time	Not applicable
Main results	16	(a) Give unadjusted estimates and, if applicable, confounder-adjusted estimates and their precision (e.g., 95% confidence interval). Make clear which confounders were adjusted for and why they were included	Yes
		(b) Report category boundaries when continuous variables were categorized	Yes
		(c) If relevant, consider translating estimates of relative risk into absolute risk for a meaningful time period	Not applicable
Other analyses	17	Report other analyses done – *e.g.* analyses of subgroups and interactions, and sensitivity analyses	Yes
**Discussion**			
Key results	18	Summarise key results with reference to study objectives	Yes
Limitations	19	Discuss limitations of the study, taking into account sources of potential bias or imprecision. Discuss both direction and magnitude of any potential bias	Yes
Interpretation	20	Give a cautious overall interpretation of results considering objectives, limitations, multiplicity of analyses, results from similar studies, and other relevant evidence	Yes
Generalisability	21	Discuss the generalisability (external validity) of the study results	Yes
**Other information**			
Funding	22	Give the source of funding and the role of the funders for the present study and, if applicable, for the original study on which the present article is based	Yes

* Give information separately for exposed and unexposed groups.

Note: An Explanation and Elaboration article discusses each checklist item and gives methodological background and published examples of transparent reporting. The STROBE checklist is best used in conjunction with this article (freely available on the Web sites of PLoS Medicine at http://www.plosmedicine.org/, Annals of Internal Medicine at http://www.annals.org/, and Epidemiology at http://www.epidem.com/). Information on the STROBE Initiative is available at http://www.strobe-statement.org.
